# A pilot study to evaluate home-based screening for the common non-communicable diseases by a dedicated cadre of community health workers in a rural setting in India

**DOI:** 10.1186/s12889-018-6350-4

**Published:** 2019-01-03

**Authors:** Partha Basu, Manoj Mahajan, Nilesh Patira, Sangita Prasad, Sushma Mogri, Richard Muwonge, Eric Lucas, Rengaswamy Sankaranarayanan, Swami Iyer, Navami Naik, Kirti Jain

**Affiliations:** 1 Screening Group, Early Detection and Prevention Section, International Agency for Research on Cancer, WHO, 150 cours Albert Thomas, 69372, Lyon, Cedex 08 France; 2 GBH Memorial Cancer Hospital, Udaipur, Rajasthan India; 3 Research Triangle Institute, International-India, Commercial Tower, Pullman Hotel Aerocity, New Delhi, India; 4 Division of Cancer Medicine, University of Texas MD Anderson Cancer Center, Houston, USA; 5 Indo-American Cancer Association, Houston, USA

**Keywords:** Non-communicable disease, screening, hypertension, diabetes, cancer, self-collection, Human Papillomavirus test

## Abstract

**Background:**

Population-based screening for the common non-communicable diseases (NCD) is recommended but is difficult to implement in the hard-to-reach areas of low resourced countries. The objective of our pilot study was to evaluate the feasibility and the efficacy of delivering NCD screening services at home by trained community health workers (CHWs). Men and women aged 30-60 years residing in rural areas of India were targeted for screening.

**Methods:**

The CHWs made home visits to educate the participants about healthy lifestyles and symptoms of common cancers and counsel the tobacco/alcohol users to quit. They measured height, weight, blood pressure (BP) and random blood sugar for all and performed oral visual examination (OVE) to screen the tobacco/alcohol users for oral cancer. For cervical cancer screening, the women themselves provided self-collected vaginal samples that the CHWs delivered to the laboratory for high-risk Human Papillomavirus (HPV) detection. The women were not screened for breast cancer but were made aware of the common symptoms and the importance of early diagnosis. Further assessment of the screen-positive individuals and the women with breast symptoms was arranged at the nearest primary health center (PHC).

**Results:**

The CHWs screened 1998 men and 4997 women from 20 villages within 6 months; the refusal rate was less than 10%. High BP and sugar were detected in 32.6% and 7.5% participants respectively; hypertension and diabetes were confirmed in 42.3% and 35% respectively among those undergoing follow-up. Obesity prevalence was only 2.4%. More than 50% men were tobacco chewers. Of the total participants, 2.6% were positive on OVE, though no oral cancer was detected among them. HPV test was positive in 8.6% women and they were triaged with visual inspection after application of acetic acid (VIA) test for treatment either by thermal ablation (same visit) or by loop excision. VIA was positive in 14% of the HPV-positive women and 56.5% of them received same day ablative treatment. The VIA-negative women were advised follow up after 1 year. No breast cancer was detected among the 0.6% women complaining of breast symptoms.

**Conclusions:**

Delivery of NCD screening services at home by trained CHWs is feasible and well-accepted by our study population.

## Background

Noncommunicable diseases (NCDs) are the leading causes of mortality across the globe, accounting for 39.5 million deaths in 2015 [1]. Government of India, as part of its commitment to reduce premature mortality from NCDs, reorganized the NCD control program in 2016 aligned to the WHO Global NCD Action Plan 2013-2020 and introduced screening for the common NCDs [2]. The program guidelines stipulate screening of males and females (age 30 years and above) for hypertension, diabetes and oral cancer at the nearest community clinics (health sub-centers) and screening of females for breast and cervical cancer at the next levels of health service delivery [primary health centers (PHC) and district hospitals]. A major challenge to implement the program is that the sub-centers are under-staffed, poorly equipped and are rarely visited by the residents [3]. Long distance, inadequate transport facilities, high travel cost and loss of wages are major barriers for the rural population to seek preventive health services at the PHCs or higher centers [4]. The problem of ensuring equitable access to preventive health care in the rural and the remote areas is not unique to India but to all the low and middle income countries (LMICs) implementing NCD screening programs [5]. The community health workers (CHWs) are well-recognized to deliver different preventive health care services at the doorsteps of the beneficiaries, especially in the LMICs. The female CHW (known as ‘Accredited Social Health Activist or ASHA’) in India works as an interface between the community and the primary health care. Each ASHA is selected from the village she caters to and usually has a target population of 1,000 to cover. She receives a performance-based incentive for promoting universal immunization, referral and escort services for reproductive and child Health and other healthcare programs.

Our pilot study aimed to evaluate a novel service delivery approach to provide the early detection facilities for the common NCDs (hypertension, diabetes, breast, cervical and oral cancers) at home by trained CHWs in a rural setting. The feasibility and efficacy of the CHW-driven service delivery model were assessed by estimating the coverage of the targeted households, the compliance of screen-positives to further evaluation, the detection rates of the targeted diseases and the positive predictive values of the tests. The study provided an opportunity to study the common risk factors for diabetes and hypertension in the rural community.

## Methods

The study, conducted between January 2017 and December 2017, was approved by the ethics committees of International Agency for Research on Cancer (IARC), France and GBH American Hospital, India.

### Study design and setting

The community-based cross-sectional study was conducted by GBH Memorial Cancer Hospital (GBHMCH) and American International Institute of Medical Sciences (AIIMS) in the Udaipur district of Rajasthan, India. The study area is one of the backward rural districts of Rajasthan due to its low literacy level, semi-arid landscape, low level of agricultural production as well as its high tribal concentration. The study was conducted in Gogunda rural block having a total population of approximately 200,000 spread across 232 villages; the population in the villages ranging from 88 to 2,787 [6]. Gogunda has four primary health centers (PHC). Private health care is almost non-existent. Total 20 villages having an adult population of approximately 12,000, belonging to Gogunda block and served by a common PHC were selected to implement the study.

### Selection and training of CHWs

Female CHWs (N=10) were recruited following the same criteria of selecting CHWs for other health programs in India. The incumbents were between 25 and 45 years of age, belonged to the local community and were educated at least up to secondary level. CHWs were trained over two weeks at GBHMCH. The training was structured to ensure both knowledge development and skill acquirement and was followed by an exit evaluation. After successful completion of training, each CHW performed the counseling and the screening procedures under direct supervision of the investigators at the GBHMCH out-patients for one week. A reorientation training was held after three months. Each CHW was provided with a kit that contained the following:

1. A flipchart to educate the study participants
2. A portable electronic scale and a measuring tape
3. A digital upper arm blood pressure (BP) monitor (Omron^TM^ Healthcare, Japan)
4. A blood glucose meter (Accucheck^TM^, Roche, Germany)
5. A torch and a packet of wooden spatula for oral visual examination
6. A prosthetic silicone breast model with a palpable nodule inside to educate the women about breast cancer early detection
7. Digene^TM^ Cervical Samplers, each containing a cervical brush and specimen transport medium

The weighing machines, BP monitors and glucometers were checked by the investigators during each monthly meeting of the CHWs. Each BP monitor was validated using a mercury sphygmomanometer. Each weighing machine was cross checked using standard weights. The blood glucose of a hospitalized patient was tested with the glucometer just before the same patient provided blood sample for estimation of blood glucose at the GBHMCH laboratory.

### Selection of study participants

Men and women between 30 and 60 years of age and residing in the selected villages were eligible. Individuals suffering from debilitating illnesses and the pregnant women were excluded. The pregnant women were advised to attend antenatal check-ups and appropriate investigations as per the reproductive health care program guidelines. The study aimed to recruit 5,000 women and 2,000 men. The sample size was empirically decided to recruit enough number of men and women to evaluate the feasibility and efficacy of the pilot interventions. We recruited higher number of women to assess the performance of the self-collected samples for HPV test at home, a novel approach in Indian setting.

The CHWs worked in groups of two and systematically visited every household of the village. In every household the eligible men and women were invited to participate in the study and a written informed consent was obtained from each of them. The CHWs revisited the household on a later date if an eligible member was not available during the initial visit. Screening of men was stopped after recruiting 2000 participants, while screening of women continued till 5000 participants were recruited.

### Procedures at home visit

Every study participant was educated by the CHWs about healthy lifestyle, the harmful effects of tobacco and alcohol and the symptoms of common cancers. The self-reported current tobacco and/or alcohol users (defined as those consumed tobacco and/or alcohol at least once in past 30 days) were counselled to quit. Weight was measured with light garments on and height was recorded without any shoes. BP was recorded with the subject sitting comfortably after at least 15 minutes of rest. If the initial recoding exceeded 140/90 mm Hg, a second measurement was obtained after 15 minutes. The lower of the two recordings was considered as final. Random blood glucose was estimated with blood sample drawn from a finger prick using sterile lancet and the displayed reading on the glucometer was recorded. A systematic oral visual examination (OVE) followed by palpation of an abnormal area was performed on every participant self-reporting to be ever tobacco and/or alcohol user. All women were made aware of the early symptoms of breast cancer and the benefits of early diagnosis. They were educated about the feel of a breast lump using the prosthetic breast model. The women provided self-collected upper vaginal sample for Human Papillomavirus (HPV) detection test. The CHW explained the steps of collection using a visual chart and the women collected the sample in the privacy of another room or toilet. At the end the CHW issued a health card documenting the test results. Participants with high BP. high glucose, abnormal OVE or breast symptoms were advised to attend the nearest PHC for further evaluation. An already known hypertensive or diabetic was advised to attend the PHC, if his/her BP or random glucose was high or if he/she was not taking medicines advised by a physician.

### HPV detection test

The cervical samples were tested for 14 most common oncogenic HPV types using the CareHPV^TM^ (Qiagen, Gaithersburg, USA) test. The test facility was set up at the GBHMCH laboratory. The CHWs deposited the samples to the laboratory once a week. Till that time, the samples were stored at room temperature. A technician was trained to run the CareHPV test following the manufacturer’s instructions. Samples with relative light unit (RLU)/cut-off ratio >1 were considered positive. The CHWs collected the reports from the laboratory during their weekly visits to GBHMCH and distributed them to the women.

### Further evaluation at PHC

We followed the referral criteria mentioned in the Indian National Guidelines for high BP (>140/90 mm) and high random blood glucose (>140 mg/dl). At the PHC, all the referred participants had BP checked by a medical officer and had fasting and/or post-prandial blood glucose estimated. Hypertension was confirmed if BP was >140/90 mm Hg up on repeat checkup. An individual with a fasting glucose of >120 mg/dl and/or a post-prandial glucose of >140 mg/dl was diagnosed as a diabetic.

Individuals with abnormal OVE or women complaining of breast symptoms were examined at the PHC by a clinician trained at GBHMCH. Further evaluation (biopsy for suspected oral premalignant lesions or mammography/ultrasound for breast abnormalities) was performed at the GBHMCH based on the clinician’s recommendations.

The HPV-positive women were examined at the PHC by a trained gynecologist who performed visual inspection of cervix after application of 5% acetic acid (VIA). Women with positive VIA test were treated by thermal-ablation after taking a punch biopsy, if the lesion was suitable for ablative treatment (type I transformation zone, lesion purely ectocervical and occupying less than 75% of the ectocervix and no suspicion of cancer). The VIA-positive women who were not eligible for ablative treatment were referred to GBHMCH for colposcopy and further management. The HPV-positive but VIA-negative women were advised repeat checkup after 1 year.

The records of each participant were maintained in an online password protected database. The project coordinators (N=2) tracked the screen positive participants and reminded them over phone, if the follow up was not completed within a month of referral.

### Statistical considerations

Statistical analysis was performed using the Stata 14.2 (StataCorp LP, Texas, USA) software. The distribution of participant socio-demographic, reproductive characteristics, tobacco and alcohol consumption, and clinical findings, overall and stratified by sex, were presented as numbers and proportions. The process measures and the intermediate outcomes were also presented as numbers and proportions. The process measures included numbers screened for hypertension, diabetes, oral, breast and cervical lesions; screen positivity; attendance for referral after an abnormality on any of the screening tests done; and treatment received. The intermediate outcomes included confirmed hypertension, confirmed diabetes, detection of oral, breast and cervical neoplasias during the further investigations. The detection rates of hypertension and diabetes were estimated as the proportion of the men and women with confirmed disease out of the total screened after exclusion of the known hypertensives or the diabetics. The positive predictive value (PPV) of the screening tests was estimated as the proportion of individuals with confirmed disease out of those undergoing diagnostic assessments. Because of the possible interaction between hypertension and diabetes, we defined an outcome with 4 categories including: participants with neither hypertension nor diabetes; those with hypertension but no diabetes; those with diabetes but no hypertension; and those with both. The last 3 categories then defined our studied endpoints. The effect of participant characteristics on the 3 endpoints was assessed using odds ratios (ORs) and their 95% confidence intervals (CIs) obtained from multinomial logistic regression models. Regression analysis models were adjusted for clustering on study village to allow for the possible correlation of within village responses, that is, the responses are independent between but not necessarily within study villages. Adjustment in the regression models was done by including all the cofactors assessed in the multivariate analysis. P for trend values were obtained to assess the dose-response effects of age, education and BMI grade in a multivariate model in which these factors were included as ordinal variables. For each endpoint, the group of participants with neither hypertension nor diabetes was used as the base outcome in the regression analyses.

## Results

Screening of 1,988 men and 4,997 women was completed within six months. Nearly 90% of the targeted households were covered; the residents of the remaining households either were not available or refused to participate. The demographic details of the participants are described in table 1. The mean age of the screened men (44 + 10 Years) was higher than that of the women (38 + 8 years) due to the local custom of men marrying younger women. The literacy rate was low with 29.2% men and 12.7% women being educated at high schools or colleges. Tobacco use was highly prevalent in men; 51.8% being current chewer of tobacco and 27.7% being current smoker. Current alcohol consumption was reported by 29.5% of the men. Rate of consumption of tobacco or alcohol was significantly less among the women. Only 2.6% women reported to be current chewer of tobacco and only 0.2% were current smoker. None of the participants ever had screening for NCDs.

**Table 1:** Participants’ characteristics by gender

Characteristics	All		Men		Women	
	n (%)		n (%)		n (%)	
Participants assessed	6,985		1,988		4,997	
Age (years)						
30-34	2,420	(34.6)	403	(20.3)	2,017	(40.4)
35-39	1,441	(20.6)	351	(17.7)	1,090	(21.8)
40-44	1,065	(15.2)	298	(15.0)	767	(15.3)
45-49	695	(9.9)	260	(13.1)	435	(8.7)
50-54	614	(8.8)	222	(11.2)	392	(7.8)
55-60	750	(10.7)	454	(22.8)	296	(5.9)
Marital status						
Unmarried	8	(0.1)	7	(0.4)	1	(0.0)
Married	6,865	(98.3)	1,963	(98.7)	4,902	(98.1)
Widowed	99	(1.4)	13	(0.7)	86	(1.7)
Separated	13	(0.2)	5	(0.3)	8	(0.2)
Age at marriage (years)						
<18	2,391	(34.2)	490	(24.6)	1,901	(38.0)
18+	4,402	(63.0)	1,423	(71.6)	2,979	(59.6)
Unknown/not applicable	192	(2.7)	75	(3.8)	117	(2.3)
Age at first child birth (years)						
<18					644	(12.9)
18+					4,208	(84.2)
Unknown/not applicable					145	(2.9)
Current residence type of house						
Thatched	3,650	(52.3)	1,150	(57.8)	2,500	(50.0)
Tiled	3,323	(47.6)	833	(41.9)	2,490	(49.8)
Concrete	11	(0.2)	4	(0.2)	7	(0.1)
Unknown	1	(0.0)	1	(0.1)	0	(0.0)
Average monthly household income (in rupees)^a^						
<5000	4,075	(58.3)	1,021	(51.4)	3,054	(61.1)
5000-9999	1,638	(23.5)	632	(31.8)	1,006	(20.1)
10000-19999	995	(14.2)	235	(11.8)	760	(15.2)
≥20000	276	(4.0)	99	(5.0)	177	(3.5)
Unknown	1	(0.0)	1	(0.1)	0	(0.0)
Religion						
Hindu	6,913	(99.0)	1,969	(99.0)	4,944	(98.9)
Muslim	54	(0.8)	14	(0.7)	40	(0.8)
Christian	15	(0.2)	3	(0.2)	12	(0.2)
Unknown	3	(0.0)	2	(0.1)	1	(0.0)
Participant’s education						
Nil	3,130	(44.8)	472	(23.7)	2,658	(53.2)
Primary	1,556	(22.3)	509	(25.6)	1,047	(21.0)
Middle	1,084	(15.5)	426	(21.4)	658	(13.2)
High school	662	(9.5)	317	(15.9)	345	(6.9)
College	552	(7.9)	264	(13.3)	288	(5.8)
Unknown	0	(0.0)	0	(0.0)	0	(0.0)
Participant’s occupation						
Unemployed/Home maker	3,676	(52.6)	72	(3.6)	3,604	(72.1)
Manual labour	1,945	(27.8)	1,014	(51.0)	931	(18.6)
Agriculture	723	(10.4)	406	(20.4)	317	(6.3)
Service	392	(5.6)	268	(13.5)	124	(2.5)
Own business	206	(2.9)	187	(9.4)	19	(0.4)
Professional	5	(0.1)	5	(0.3)	0	(0.0)
Retired	37	(0.5)	35	(1.8)	2	(0.0)
Unknown	1	(0.0)	1	(0.1)	0	(0.0)
Tobacco chewing status						
Never	5,752	(82.3)	901	(45.3)	4,851	(97.1)
Current^a^	1,160	(16.6)	1,029	(51.8)	131	(2.6)
Past	73	(1.0)	58	(2.9)	15	(0.3)
Tobacco smoking status						
Never	6,343	(90.8)	1,360	(68.4)	4,983	(99.7)
Current^a^	560	(8.0)	550	(27.7)	10	(0.2)
Past	82	(1.2)	78	(3.9)	4	(0.1)
Alcohol consumption status						
Never	6,266	(89.7)	1,302	(65.5)	4,964	(99.3)
Current^a^	611	(8.7)	587	(29.5)	24	(0.5)
Past	108	(1.5)	99	(5.0)	9	(0.2)
^a^Rupees 100 ≈ US$ 1.4						

^a^Current tobacco or alcohol users were defined as those consumed tobacco or alcohol at least once in past 30 days

Results of the different screening tests are listed in Table 2. High BP (>140/90 mm) was recorded in 48.0% men and 26.4% women. Random glucose was >140 mg/dl in 7.5% participants (men 10.7% and women 6.2%). The prevalence of obesity was low in the rural and mostly indigent population. Only 2.4% of the participants were obese and 8.7% were overweight.

**Table 2:** Results of the screening tests by gender

	All participants		Men		Women	
	n (%)		n (%)		n (%)	
Participants screened	6,985		1,988		4,997	
Blood pressure status (mm Hg)						
Not high	4,578	(65.5)	975	(49.0)	3,603	(72.1)
High (140+/90+) at screening	2,275	(32.6)	955	(48.0)	1,320	(26.4)
Known hypertensive	132	(1.9)	58	(2.9)	74	(1.5)
Blood sugar levels (mg/dl)						
Not high	6,395	(91.6)	1,734	(87.2)	4,661	(93.3)
High (140+ mg/dl) at screening	522	(7.5)	213	(10.7)	309	(6.2)
Known diabetic	68	(1.0)	41	(2.1)	27	(0.5)
BMI grade (Kg/m^2^)						
Underweight (<18.5)	1,349	(19.3)	284	(14.3)	1,065	(21.3)
Normal (18.5-<25.0)	4,864	(69.6)	1,314	(66.1)	3,550	(71.0)
Overweight (25.0-<30.0)	606	(8.7)	302	(15.2)	304	(6.1)
Obese (30.0+)	166	(2.4)	88	(4.4)	78	(1.6)
Oral visual inspection findings						
Normal	6,803	(97.4)	1,828	(92.0)	4,975	(99.6)
Abnormal	182	(2.6)	160	(8.0)	22	(0.4)
HPV results						
Negative					4,566	(91.4)
Positive					429	(8.6)
Breast symptoms						
Normal					4,967	(98.3)
Abnormal					28	(0.6)

*HPV* human papilloma virus, *BMI* body mass index (weight/height^2^)

OVE was abnormal in 8.0% men and 0.4% women. HPV test was positive among 8.6% women. None of the women participating in the study refused or failed to collect the vaginal samples. Only 32 samples were found to be inadequate due to leakage of the contents during transport; 30 of them could be re-collected from the women. Only 28 (0.6%) women complained of breast lump or nipple discharge while they were visited by the CHWs.

The results of further assessment of the participants referred for high BP and sugar are given in Table 3. Among the 2,275 referrals for high BP, 1,475 (64.8%) were further evaluated and of them 624 (42.3%) were confirmed to be hypertensives. Out of the 522 participants with high sugar at screening, 412 (78.9%) attended the PHC and 144 (35.0%) were confirmed to be diabetic. The detection rate of hypertension in the screened population was 9.3% (636/6,853) and that of diabetes was 2.4% (167/6,917). The PPVs of screening for BP and blood sugar to detect the disease were 42.3% and 35.0% respectively.

**Table 3:** Participant screening test results and follow-up investigations

Baseline characteristics	Assessed at baseline	With further investigations					
		Assessed		Confirmed Hypertension		Confirmed Diabetes	
	n	n (%)		n (%)		n (%)	
*All participants*							
Participants screened	6,985	1,824	(26.1)	665	(36.5)		
Blood pressure status (mm Hg)							
Not high	4,578	257	(5.6)	12	(4.7)		
High (140+/90+) at screening	2,275	1,475	(64.8)	624	(42.3)		
Known hypertensive	132	92	(69.7)	29	(31.5)		
Blood pressure status (mm Hg)							
Not high	4,578	257	(5.6)	12	(4.7)		
High (140-159/90+) at screening	1,998	1,243	(62.2)	449	(36.1)		
Very high (160+/90+) at screening	277	232	(83.8)	175	(75.4)		
Known hypertensive	132	92	(69.7)	29	(31.5)		
Blood sugar levels (mg/dl)							
Not high	6,395	1,356	(21.2)			23	(1.7)
High (140+ mg/dl) at screening	522	412	(78.9)			144	(35.0)
Known diabetic	68	56	(82.4)			36	(64.3)
Blood sugar levels (mg/dl)							
<126	5,746	1,192	(20.7)			22	(1.8)
126-139	649	164	(25.3)			1	(0.6)
140-159	325	280	(86.2)			70	(25.0)
160-179	90	55	(61.1)			17	(30.9)
180-199	52	25	(48.1)			15	(60.0)
200+	55	52	(94.5)			42	(80.8)
Known diabetic	68	56	(82.4)			36	(64.3)
BMI grade (Kg/m^2^)							
Underweight (<18.5)	1,349	303	(22.5)	89	(29.4)	27	(8.9)
Normal (18.5-<25.0)	4,864	1,168	(24.0)	434	(37.2)	122	(10.4)
Overweight (25.0-<30.0)	606	267	(44.1)	105	(39.3)	38	(14.2)
Obese (30.0+)	166	86	(51.8)	37	(43.0)	16	(18.6)

*BMI* body mass index (weight/height^2^)

VIA was performed on 76.5% (328/429) HPV-positive women. Only 46 women (14.0%) were VIA-positive and were biopsied. Histopathology reports were normal in 26; CIN 1 in 9; CIN 2 in 6; CIN 3 in 4; and invasive squamous cell cancer in 1. Thermal-ablation was performed on all 26 women eligible for ablative treatment. Women with CIN2+ diagnosis on histopathology and not treated by thermal-ablation were treated at GBHMCH.

A single case of fibroadenoma was detected among the 28 women with breast symptoms. Rest did not have any significant abnormalities on clinical examination and no further investigations were advised. None of the 181 men or women examined for positive OVE had any growth or ulcer in the oral cavity. They were counseled to quit tobacco and alcohol.

Table 4 describes the screening outcome by participants’ socio-demographic characteristics, BMI, tobacco and alcohol consumption. Table 5 presents the independent effect (multivariate analysis) of these factors on the risk of the three endpoints, that is, being hypertensive but not diabetic; being diabetic but not hypertensive; and being both hypertensive and diabetic. A significantly increased risk of hypertension was observed with increasing age (risks estimates ranging from 1.2 to 3.5-fold compared to those aged 30-34 years) with a significant dose-response relationship (*p*-value for trend <0.001). The risk of hypertension was significantly higher among the overweight (OR=2.0; 95% CI=1.5-2.7) and the obese (OR=3.0; 95% CI=2.3-3.9) population compared to those having normal weight, with a significant dose response relationship (p-value for trend=<0.001). The ever consumers of alcohol also had a higher risk (OR=1.4; 95% CI=0.9-2.0) compared to the never consumers.

**Table 4:** Distribution of hypertension and diabetes status at baseline by participants’ characteristics

characteristics	Neither hypertensive nor diabetic	Hypertensive but not diabetic	Diabetic but not hypertensive	Both hypertensive and diabetic
Participants assessed	5,194	681	148	84
Age (years)				
30-34	2,009	138	24	10
35-39	1,129	108	24	7
40-44	773	110	27	9
45-49	471	83	19	12
50-54	382	100	17	16
55-60	430	142	37	30
Sex				
Men	1,231	282	81	46
Women	3,963	399	67	38
Education				
Nil	2,286	339	63	36
Primary	1,184	128	27	23
Middle	824	91	26	12
High school	484	71	18	4
College	415	52	14	9
BMI grade (Kg/m^2^)				
Underweight (<18.5)	1,072	89	23	6
Normal (18.5-<25.0)	3,692	452	90	46
Overweight (25.0-<30.0)	346	105	24	24
Obese (30.0+)	84	35	11	8
Chewing status				
Never	4,431	514	109	60
Ever	763	167	39	24
Smoking status				
Never	4,802	581	130	70
Ever	392	100	18	14
Alcohol consumption status				
Never	4,780	570	130	71
Ever	414	111	18	13

*BMI* body mass index (weight/height^2^)

**Table 5:** Effect of participant characteristics on hypertension and/or diabetes status at baseline

Participant characteristics	Endpoint as hypertensive but not diabetic (excluding participants with diabetes)				Endpoint as diabetic but not hypertensive (excluding participants with hypertension)				Endpoint as both with hypertensive and diabetic (excluding participants with only hypertension and those with only diabetes)			
	OR (95% CI)				OR (95% CI)				OR (95% CI)			
	*Crude analysis*											
Age (years)												
30-34	1.0				1.0				1.0			
35-39	1.4	(1.0	-	2.0)	1.8	(1.0	-	3.0)	1.2	(0.4	-	4.0)
40-44	2.1	(1.6	-	2.7)	2.9	(1.9	-	4.6)	2.3	(1.1	-	4.9)
45-49	2.6	(1.7	-	3.9)	3.4	(1.9	-	6.2)	5.1	(2.4	-	10.8)
50-54	3.8	(2.8	-	5.2)	3.7	(2.4	-	5.8)	8.4	(3.7	-	19.3)
55-60	4.8	(3.7	-	6.3)	7.2	(4.6	-	11.4)	14.0	(6.0	-	32.7)
Sex												
Men	1.0				1.0				1.0			
Women	0.4	(0.3	-	0.6)	0.3	(0.2	-	0.4)	0.3	(0.1	-	0.5)
Education												
Nil	1.0				1.0				1.0			
Primary	0.7	(0.6	-	0.9)	0.8	(0.5	-	1.3)	1.2	(0.7	-	2.1)
Middle	0.7	(0.6	-	0.9)	1.1	(0.7	-	1.9)	0.9	(0.6	-	1.5)
High school	1.0	(0.7	-	1.3)	1.3	(0.7	-	2.6)	0.5	(0.2	-	1.7)
College	0.8	(0.6	-	1.2)	1.2	(0.6	-	2.6)	1.4	(0.5	-	3.8)
BMI grade (Kg/m^2^)												
Underweight (<18.5)	0.7	(0.5	-	1.0)	0.9	(0.6	-	1.3)	0.4	(0.2	-	1.0)
Normal (18.5-<25.0)	1.0				1.0				1.0			
Overweight (25.0-<30.0)	2.5	(1.8	-	3.5)	2.8	(1.8	-	4.5)	5.6	(2.6	-	11.9)
Obese (30.0+)	3.4	(2.4	-	4.9)	5.4	(3.2	-	8.9)	7.6	(4.2	-	14.1)
Chewing status												
Never	1.0				1.0				1.0			
Ever	1.9	(1.4	-	2.5)	2.1	(1.3	-	3.3)	2.3	(1.4	-	3.7)
Smoking status												
Never	1.0				1.0				1.0			
Ever	2.1	(1.5	-	3.0)	1.7	(0.9	-	3.0)	2.5	(1.3	-	4.6)
Alcohol consumption status												
Never	1.0				1.0				1.0			
Ever	2.2	(1.6	-	3.1)	1.6	(0.9	-	2.7)	2.1	(1.3	-	3.3)
	Adjusted analysis^a^											
Age (years)												
30-34	1.0				1.0				1.0			
35-39	1.3	(0.9	-	1.8)	1.6	(0.9	-	2.7)	1.1	(0.3	-	3.6)
40-44	1.7	(1.3	-	2.3)	2.4	(1.6	-	3.7)	1.8	(0.8	-	4.1)
45-49	2.0	(1.3	-	3.0)	2.5	(1.4	-	4.5)	3.8	(1.5	-	9.1)
50-54	3.0	(2.3	-	3.8)	2.7	(1.8	-	4.2)	5.7	(2.0	-	16.0)
55-60	3.5	(2.6	-	4.6)	4.6	(2.9	-	7.3)	9.5	(3.6	-	24.9)
*p*-value for trend	<0.001				<0.001				<0.001			
Sex												
Men	1.0				1.0				1.0			
Women	0.7	(0.4	-	1.1)	0.3	(0.2	-	0.5)	0.5	(0.2	-	1.1)
Education												
Nil	1.0				1.0				1.0			
Primary	0.7	(0.6	-	0.8)	0.7	(0.5	-	1.1)	1.2	(0.7	-	2.1)
Middle	0.8	(0.6	-	1.0)	0.9	(0.6	-	1.6)	0.9	(0.5	-	1.7)
High school	0.9	(0.7	-	1.1)	0.9	(0.5	-	1.7)	0.4	(0.1	-	1.3)
College	0.8	(0.5	-	1.2)	0.8	(0.4	-	1.7)	1.1	(0.3	-	4.0)
*p*-value for trend	0.597				0.844				0.869			
BMI grade (Kg/m^2^)												
Underweight (<18.5)	0.7	(0.5	-	0.9)	1.0	(0.7	-	1.5)	0.5	(0.2	-	1.0)
Normal (18.5-<25.0)	1.0				1.0				1.0			
Overweight (25.0-<30.0)	2.0	(1.5	-	2.7)	2.1	(1.4	-	3.1)	4.2	(1.9	-	9.3)
Obese (30.0+)	3.0	(2.3	-	3.9)	4.2	(2.7	-	6.3)	7.0	(3.8	-	12.8)
*p*-value for trend	<0.001				<0.001				<0.001			
Chewing status												
Never	1.0				1.0				1.0			
Ever	1.1	(0.8	-	1.6)	1.0	(0.5	-	1.7)	1.2	(0.6	-	2.1)
Smoking status												
Never	1.0				1.0				1.0			
Ever	0.9	(0.8	-	1.2)	0.6	(0.4	-	1.0)	0.8	(0.4	-	1.4)
Alcohol consumption status												
Never	1.0				1.0				1.0			
Ever	1.4	(0.9	-	2.0)	0.7	(0.4	-	1.3)	1.0	(0.5	-	1.9)

*OR* odds ratio, *CI* confidence interval, *BMI* body mass index (weight/height^2^); ^a^All variable included in the multivariate regression model

For the diabetes endpoint, the risk significantly increased with increasing age (ORs ranging from 1.6 to 4.6 compared to those aged 30-34 years) with a significant dose-response relationship (*p*-value for trend <0.001). The risk was significantly higher in the overweight participants (OR=2.1; 95% CI=1.4-3.1) and the obese (OR=4.2; 95% CI=2.7-6.3) compared to those having normal weight, again with a significant dose response relationship (p-value for trend=<0.001).

## Discussion

Detection of a large number of previously unknown hypertensives and diabetics, successful screening and treatment of women for cervical neoplasias, low refusals to screening and high compliance of the screen-positives to further assessment validate our model of providing NCD screening services at home by the CHWs. However, a major challenge to scale up such an approach is that the CHWs in the existent health services are engaged in providing various other services, mostly related to reproductive and child health. While the CHWs in our study could dedicate full time to the screening activities, the CHWs in the routine health services already over-burdened with many health programs will have burn out effects. It is important for the health services to make some vertical investments and build a dedicated work force providing NCD services. Tamilnadu is the only state in India that has been able to successfully implement mass-scale NCD screening, supported initially by the World Bank. The program invested heavily in engaging and training dedicated nurses, multi-purpose health workers and auxiliary nurse midwives, not only to provide NCD services but also for community mobilization, record maintenance and data management [7].

The access to the primary care in the rural areas in the LMICs is limited by a combination of factors such as poor physical infrastructure, lack of trained providers, inadequate financing and scarcity of transport. The facilities designated to provide the basic care at the community level are often under-staffed and under-equipped and have low uptake of the services due to sub-optimal quality. The Family Health Survey (2015-16) in Rajasthan, India (where our study was implemented) reported that only 3% of the rural households visited the sub-centres during any illness [8]. While people may be ready to accept the extra cost and hardships and loss of wages to reach the higher centres situated far away during sickness, the apparently healthy individuals targeted for screening are unlikely to bear the cost and the hardships for the preventive services [9].

The problem of accessing health care is even more complex for the women. Women in rural India like in many other LMICs are the victims of health inequity as a consequence of discriminatory beliefs and practices, lack of awareness (knowledge of women, their families and the health care providers about the existence of a health problem) and acknowledgement (recognition that something should and can be done about the health problem) [10]. The NCD screening program to succeed in LMICs need to deliver the services at the doorsteps of the beneficiaries. To deliver NCD prevention and early detection services at the doorsteps, the CHWs are the most key members of the healthcare team.

CHWs have been successfully utilized in many countries to facilitate access to care, increase quality of service and improve healthcare outcomes [11]. A systematic review demonstrated that the task-shifting from physicians to health workers, with appropriate health system re-structuring is viable as well as cost-effective in improving access to healthcare for NCDs in the LMICs [12]. While in other studies, the utilization of CHWs has been limited to screening for hypertension and diabetes, we have demonstrated how the cancer early detection services can also be delivered by them at the doorsteps. The recently launched WHO guideline on health policy and system support to optimize CHW programs highlights the evolving role of CHWs over time with changes in the epidemiological profile of the diseases and the requirements of the health system [13]. The services of the CHWs need to be aligned to the growing global burden of the NCDs and it may be worthwhile to consider building a separate cadre of ‘NCD workers’ to improve the quality and reach of the NCD control services in the community.

Our approach to screening for oral, breast and cervical cancers was different from the recommendations of the Indian guidelines. The Indian guidelines recommend oral screening for all, breast cancer screening by clinical breast examination (CBE) and cervical cancer screening by VIA. We limited OVE to the ever-users of tobacco/alcohol, since oral cancer screening is ineffective in the non-users [14]. We made the women aware of early symptoms of breast cancer rather than screening them with CBE. CBE screening has demonstrated significant downstaging of breast cancer but there is inadequate evidence for its impact on mortality reduction [15, 16]. We followed the early diagnosis approach (breast awareness combined with ready access to diagnosis and treatment) that has been recommended by World Health Organization as simple, effective and feasible in all LMICs [17]. We used HPV detection test that has proved to be significantly more efficacious than VIA and allows screening to be performed at home [18, 19].

Ours was the first Indian study to perform HPV testing of self-sampled vaginal specimens collected at home setting. In the earlier studies the women provided the samples in hospital-based or community-based clinics [20, 21]. The HPV positivity (8.6%) was comparable to the test positivity (7.7%) reported in the other Indian study that used CareHPV^TM^ to test self-collected samples. The compliance of the women was high, there were very few operational problems and the technology transfer was easy.

The comparability of our screening test results with other Indian studies and the high positive predictive values (PPV) of further assessment to detect hypertension or diabetes (42.3% for high BP and 35.0% for high sugar) reinforce the validity of our approach. The Integrated Disease Surveillance Project (2007-2008) by the Government of India reported high BP ranging from 18% to 26% among rural population (age 15-64 years) across different provinces [22]. Another nationally representative, population-based study screened a total of 1.3 million Indian adults (>18 years) for hypertension and diabetes [23]. The reported prevalence of hypertension (25.3%) and diabetes (7.5%) were similar to the values we observed.

If high referral rate for high BP (32.6%) is a concern, the rate could be drastically reduced by modifying the referral threshold to 160/90 mm, which also resulted in significant improvement in the PPV. In our study only 4.0% (277/6,985) participants had BP >160/90 mm at screening, and the PPV of high BP at screening at this threshold was 75.4% for the final diagnosis of hypertension. Benefit of revising the referral threshold has been observed by earlier studies and may be considered for population screening [24].

There are several unanswered questions in our study, especially related to the integration of the CHW-driven strategy in the larger program, e.g. improving compliance to further assessment, monitoring and quality assurance mechanism and cost-effectiveness in a scaled-up program. The missing follow-up data could potentially alter the test characteristics. We did not assess the impact of awareness or study the adherence to the recommended treatment, which are important factors for success of such a program. We have conducted a separate qualitative study to identify the structural barriers to implement CHW-driven NCD control in the study setting and also the knowledge, attitude and perceptions of the men and the women that could influence uptake of services. The results will be published separately. The generalisability of the study findings in India may be questioned given the heterogeneity in population characteristics and the health system organization. Considering the facts that 75% of the Indian populations still live in villages with problems in accessing health care, the CHWs are integral part of the health work force and have high acceptance in the community, the population has limited awareness of preventive health care - the CHW-driven approach has high relevance across the country, especially in the context of the revamped national NCD control program. Further evaluation studies are needed with an appropriate ‘standard of care control’ to quantify the benefits and harms of our approach, with an emphasis on patient-relevant long-term outcomes such as morbidity and mortality reduction and also cost-effectiveness.

## Conclusions

To conclude, our pilot study demonstrated the feasibility of delivering NCD prevention and screening services at home by CHWs and the high acceptance of the services at least among our study population. The effectiveness of such an approach and the implications to the health services need to be carefully evaluated further.

## Abbreviations

BP: Blood pressure
CBE: Clinical breast examination
CHWs: Community health workers
Cis: Confidence intervals
GBHMCH: GBH Memorial Cancer Hospital
HPV: Human Papillomavirus
IARC: International agency for research on cancer
LMICs: Low and middle income countries
NCDs: Noncommunicable diseases
ORs: Odds ratios
OVE: Oral visual examination
PHC: Primary health center
PPV: Positive predictive values
VIA: Visual inspection with acetic acid
